# Community-Based Household Survey Among Children Aged 5-18 to Identify Disability Burden and Health-Related Quality of Life Following Road Traffic Accidents in Ujjain, India

**DOI:** 10.7759/cureus.100786

**Published:** 2026-01-04

**Authors:** Ashish Pathak, Davendra Baghel, Jetendra Jat

**Affiliations:** 1 Department of Global Public Health, Health Systems and Policy, Karolinska Institute, Stockholm, SWE; 2 Department of Pediatrics, Ruxmaniben Deepchand (RD) Gardi Medical College, Ujjain, IND

**Keywords:** adolescents, children, disability burden, euroqol five-dimension questionnaire, health-related quality of life, road traffic accidents

## Abstract

Introduction: The objective of the study was to report the health-related quality of life (HRQoL) following road traffic accidents (RTAs) among children.

Methods: A community-based survey using the Hindi version of the European Quality-of-Life five-dimension questionnaire (EQ-5D-5L) was conducted to collect data from the community. The survey included 2,620 households from urban and rural areas of Ujjain, India.

Results: From these households, 229 children aged 5-18 years with a history of RTA in the past one year were identified, with 27%, 63%, and 10% reporting mild, moderate, and severe injuries, respectively, based on the length of hospitalization. Motorcycles, bicycles, and pedestrians constituted most RTAs. Helmet use was low (12%). EQ-5D-5L revealed that the most severe and extreme problem was pain and discomfort, whereas the least severe problem was usual activity and self-care. The most common (65%) injuries were either abrasion or fracture and dislocation. EQ-5D-5L severity index was maximum (mean 72) for lower extremity injuries.

Conclusions: The results highlight the feasibility and importance of using EQ-5D-5L to measure HRQoL in children following RTAs in low-resource settings. Pain/discomfort and lower extremity injuries significantly impair the quality of life, even one year after injury. The high burden from two-wheeler-related injuries and extremely low helmet use underscore the urgent need for creating awareness of road safety interventions, stricter enforcement of helmet laws, and child-focused rehabilitation programs in India.

## Introduction

In 2023, road traffic accidents (RTAs) were the 11th leading cause of death globally [1]. All injuries, including RTA-related deaths, are avertable, yet they cause death and disabilities at all ages [2]. In India, deaths due to RTA are underreported [3]. Despite this, RTAs are the leading cause of mortality among the 15-39-year age group in India [3]. Disabilities in children can lead to destitution for the family and change the life of the affected child and family. Major RTAs lead to missed school and education, possible future unemployment, social rejection, and psychological stress [2]. Children under 18 years constitute over 8% of all age groups experiencing RTA-related deaths in India. Male participants under 18 years were found to be disproportionately affected by RTIs [4].

There is a paucity of global and Indian community-based studies reporting the RTA disability burden and health-related quality of life (HRQoL) following RTA in children [5]. In the absence of data on RTA and the resulting disabilities, policymakers cannot plan appropriate resource allocations to mitigate RTA [1]. This critical gap in understanding the full societal and individual impact of pediatric injuries, particularly in low- and middle-income nations, impedes effective preventative strategies and the provision of adequate clinical care [5].

Furthermore, existing research on pediatric HRQoL postinjury often focuses on long-term outcomes, overlooking the critical short-term impacts that significantly influence recovery trajectories and necessitate tailored interventions [6,7]. The broader implications of these nonfatal outcomes, encompassing functional limitations, psychobehavioral alterations, and cognitive deficits, are often underemphasized, especially in the context of traffic-related injuries [7,8]. The World Health Organization defines road traffic injuries as "fatal or nonfatal injuries incurred as a result of a road traffic crash," highlighting the broad spectrum of adverse outcomes [9]. These injuries disproportionately affect children in low- and middle-income countries, where factors such as inadequate road safety infrastructure and limited access to healthcare exacerbate their severity and long-term impact [10]. RTAs represent India's sixth leading cause of mortality, imposing substantial physical, psychosocial, and economic burdens on individuals within productive age groups [11]. This elevated burden underscores the critical importance of understanding and addressing the long-term consequences of these incidents, particularly the disability burden and HRQoL among affected populations [12]. Therefore, the objective of this study was to report the HRQoL following RTA among children.

This study endeavors to fill this lacuna by examining the immediate and intermediate HRQoL outcomes among children in Ujjain, India, following RTAs, thereby providing data crucial for evidence-based policy formulation and targeted healthcare interventions. This research specifically addresses the unmet need for comprehensive data on childhood injury between 5 and 15 years of age for outcomes beyond mortality in resource-constrained settings, where unintentional injuries contribute significantly to childhood morbidity [4].

This article was previously posted to the medRxiv preprint server on April 27, 2020.

## Materials and methods

Study site, settings, and sampling method

This cross-sectional, community-based survey was conducted from January 1, 2020, to March 31, 2020, in seven villages and 10 urban slums of Ujjain district and city, respectively, using the World Health Organization injury survey methodology [13]. After listing, mapping, and a house-to-house survey of 2,620 households with 6,898 children, 229 children and adolescents aged 5-18 years with a history of RTA in the past year were identified and recruited to the study after obtaining signed informed consent from the household head. Three trained study assistants either asked the child/adolescent to self-administer the European Quality-of-Life (EuroQol) five-dimension questionnaire (EQ-5D-5L) or conducted a face-to-face interview to complete the same. The female head of the household was interviewed to collect the demographic details of the household, RTA details like the type and anatomical distribution of injury, injury mechanism, and safety compliance. Two team leaders supervised the data collection.

Sample size

Sample size was calculated using Stata 16 (StataCorp LLC, College Station, TX), based on an Indian study reporting severe injury in 15% of participants and mild injury in one-third [5]. The calculation detected a ≥15% difference around a proportion of 0.15 (90% power; two-sided α = 0.05), yielding 174 as the minimum. Accounting for 20% refusal, the target increased to 209. Ultimately, 229 participants were included in the study, exceeding the calculated target and providing a robust sample for analysis. The standard formula for calculating the sample size (n) per group for comparing two proportions is as follows:

$n = \frac{(Z_{\alpha/2} + Z_{\beta})^2 \left[ p_1(1 - p_1) + p_2(1 - p_2) \right]}{(p_1 - p_2)^2}$

where n is the required sample size per group, Z_α/2_ is the critical value for the significance level (1.96 for α = 0.05), and Z_β_ is the critical value for the power (90%). For 90% power, this is 1.28. p_1_ is the baseline proportion (0.15 or 15%) and p_2_​ is the anticipated proportion (0.30, representing a "15% difference" from the baseline). For a two-sided test, this is 1.96. (p_1​_-p_2_​) is the minimum detectable difference between proportions or the effect size = 0.15.

Data collection and management

The data on the details of RTAs in the last year, such as the type of accidents, vehicle details, and duration of hospitalization, were collected on forms and entered in Epi Info 7 (7.1.3, CDC Atlanta, Georgia, USA) after coding. The analysis was performed using Stata 16.

Ethical considerations

The study employed a simple random sampling strategy to recruit participants. The research protocol adhered strictly to the ethical principles outlined in the Declaration of Helsinki. Ethical approval was granted by the Institutional Ethics Committee of Ruxmaniben Deepchand Gardi Medical College, Ujjain (approval number: 94-A/2019). Throughout the data collection process, rigorous adherence to ethical guidelines was maintained to ensure the dignity, well-being, and confidentiality of the participants. Informed written consent was obtained from the mothers of the children, while assent was secured from all participants aged above seven years. Both consent and assent were documented in the local language, Hindi.

Data analysis

Descriptive statistics were utilized to characterize the study population and RTA characteristics, while inferential statistics, specifically chi-square tests and t-tests, were employed to explore associations between demographic variables, injury patterns, and HRQoL outcomes.

Outcome measure

HRQoL was assessed using the Hindi version of the EQ-5D-5L questionnaire (five-level, self-administered, officially translated by EuroQol) [14]. Children unable to self-complete it underwent a face-to-face interview version. As EQ-5D-5L lacked validation for Indian children at the time of the study, interview responses were double-scored, yielding excellent interrater reliability.

The EQ-5D-5L includes five dimensions: mobility, self-care, usual activities, pain/discomfort, and anxiety/depression. Each dimension is rated from level 1 (no problems) to 5 (extreme problems), creating a five-digit health state profile (11111-55555).

We summarized results with a severity index: sum dimension levels, subtract 5, and multiply by 5 (range: 0-100). Means and 95% confidence intervals (CIs) were reported. Injury severity was classified by length of hospital stay (LOS) as mild, moderate, or severe, a proxy for injury extent and resource use. This classification, though a proxy, offers a standardized approach to contextualizing the HRQoL outcomes within the broader framework of injury impact and healthcare burden [15].

## Results

Of the 229 children in the study, 167 (73%) were boys, and 62 (27%) were girls. The mean (± SD) age of children was 12 (±4) years (boys 12 (±4) and girls 11 (±4)). A total of 62 (27%) children reported mild injury, 144 (63%) moderate, and 23 (10%) severe injury, based on LOS.

In the study population, motorcycle riders constituted the largest group of victims (37%, n = 84), followed by an equal distribution of pedestrians and bicycle riders (21% each, n = 49). A critical gender-specific risk was identified among female pillion riders, where dupatta entanglement was responsible for 75% (n = 15/20) of their accidents. While collisions with objects (51%, n = 141) and vehicle slipping (39%, n = 88) were the primary crash mechanisms, nearly one-third of incidents involved a four-wheeler (29%, n = 60). Despite these risks, safety adherence was poor, with helmet usage documented in only 12% (n = 10/84) of motorcycle users (Table 1).

**Table 1:** Distribution of injury characteristics, crash mechanism, and safety compliance (n = 229) ^*^Percentages for crash mechanisms and involved parties may not sum to 100% due to multiple responses per incident ^a^The denominator consists only of female pillion riders (n = 20) ^b^The denominator consists only of motorcycle riders (n = 84)

Category	Variable	n (%)
Role of the victim	Motorcycle rider	84 (37)
	Bicycle rider	49 (21)
	Pedestrian	49 (21)
	Pillion rider (motorbike/bicycle)	47 (20)
Crash mechanism^*^	Collision with an object	141 (51)
	Vehicle slipping/skidding	88 (39)
Involved parties	Four-wheeler (car, truck, and van)	60 (29)
	Other motorcycle	51 (22)
Specific risk factors	Dupatta (scarf) entanglement^a^	15 (75)
Safety compliance	Helmet used among motorcycle riders^b^	10 (12)

Of the total sample (n = 229), 50 children (22%) completed the EQ-5D-5L questionnaire independently, while 179 children (79%) completed it through a face-to-face interview. The disability level and dimensional distribution according to EQ-5D-5L exhibit that most children had level 2 (38%) (slight problem) or level 3 (26%) mobility (moderate problem). Most severe (level 4) and extreme (level 5) problems were seen in the pain or discomfort dimension (Table 2), whereas the least problems (levels 1 and 2) were seen in usual activity and self-care.

**Table 2:** EuroQol five-dimension questionnaire frequencies and proportions reported by dimension and level n: number of children included in the study; %: column percentage; EuroQol: European Quality of Life

Parameter	Mobility, n = 229 (%)	Self-care, n = 229 (%)	Usual activities, n = 229 (%)	Pain/discomfort, n = 229 (%)	Anxiety/depression, n = 229 (%)
Level 1 (no problems)	142 (62)	156 (68)	165 (72)	92 (40)	133 (58)
Level 2 (slight problems)	43 (19)	34 (15)	23 (10)	57 (25)	60 (26)
Level 3 (moderate problems)	25 (11)	23 (10)	18 (8)	48 (21)	18 (8)
Level 4 (severe problems)	11 (5)	9 (4)	14 (6)	18 (8)	14 (6)
Level 5 (extreme problems)	8 (3)	7 (3)	9 (4)	14 (6)	4 (2)

The distribution of injury types and their anatomical locations is shown in Table 3.

**Table 3:** The distribution of types of injuries and their anatomical site in 229 children after road traffic accidents identified in the study %: column percentage; n: number of injuries

Parameter	Total, n = 615 (%)	Abrasions, n = 271 (%)	Fractures and dislocations, n = 130 (%)	Cuts, contusion, and laceration, n = 146 (%)	Muscular injury, n = 32 (%)	Internal organ injury, n = 25 (%)	Crush injury and amputation, n = 11 (%)
Head, face, and neck	85 (14)	29 (11)	2 (1)	34 (23)	2 (6)	17 (68)	1 (9)
Shoulder, back, and abdomen	35 (5)	7 (2)	10 (8)	6 (4)	3 (10)	8 (32)	1 (9)
Upper extremities	127 (21)	62 (23)	26 (20)	27 (19)	9 (28)	0 (0)	3 (27)
Lower extremities	368 (60)	173 (64)	92 (71)	79 (54)	18 (56)	0 (0)	6 (55)

The two most common injuries following RTA were abrasions along with fractures and dislocations, whereas the least common were crush or nerve injuries and amputation.

Table 4 illustrates the mean and 95% CI of the severity index. The maximum mean value of the severity index was for lower limb injuries (mean = 72), followed by the shoulder, back, and abdomen (mean = 34). The mean severity indices for mild, moderate, and severe disease based on LOS were 18, 22, and 68, respectively.

**Table 4:** The mean and its 95% confidence interval of sum-score severity index according to the anatomical site of injury after road traffic accident RTI: road traffic injury; CI: confidence interval

Parameter	RTI	Severity index	
	n = 615	Mean	95% CI
Lower extremity	85	72	66-77
Upper extremity	35	27	20-34
Head, face, and neck	127	27	18-36
Shoulder, back, and abdomen	368	34	0-68

Intraclass correlation coefficient

The intraclass correlation coefficient (ICC; k raters) was 0.92 (95% CI: 0.89-0.95), suggesting excellent interrater reliability. The ICC for test-retest reliability was 0.88 (95% CI = 0.82-0.93), indicating good to excellent temporal stability. In the present study, the EQ-5D-5L demonstrated good internal consistency with a Cronbach’s alpha of 0.82.

## Discussion

To the best of our knowledge, this is the first Indian study among children aged 5-18 years reporting HRQoL as measured by EQ-5D-5L from a community-based survey. The EQ-5D-5L instrument has been used in some studies performed in adults to assess QOL [15]. The present study proves that RTAs lead to loss of health and human capital among children, which could be averted with improved safety and prevention programs and ensuring access to care resources [1]. The Government of India has increased fund allocation toward “Road Safety” [3]. However, research funds for identifying evidence-based road safety interventions must be increased to reduce the Indian RTA burden. The substantial postpandemic decline in cases, approximately 60%, suggests a potential correlation between reduced mobility and a decreased incidence of childhood RTAs, though further research is needed to delineate specific causative factors [16]. This decline aligns with observations of decreased fracture rates in children during lockdown periods, suggesting a broader impact of restricted activity on injury epidemiology [17].

In the present study, RTAs exhibited a male predilection. One reason for such male predilection could be the patriarchal nature of Indian society, where boys are allowed more autonomy compared with girls [18]. Another reason could be higher risk-taking behavior by boys, but this was not directly studied [4]. This trend is consistently observed in trauma epidemiology, where male children frequently show higher rates of injury from external causes, including RTAs and other mechanisms such as falls and assaults [19]. Furthermore, studies in developing nations, including India, highlight that unintentional pediatric trauma, encompassing both falls and RTAs, places a significant burden on healthcare systems with limited resources [19]. These injuries often result in prolonged hospital stays, significant financial strain on families, and long-term disabilities, underscoring the necessity for robust preventive strategies and enhanced trauma care infrastructure [15].

In the present study, motorbikes were the most common cause of RTA, followed by bicycle pillion riders. Large Indian data also point to similar injury patterns in children [3]. Injury among two-wheeler pillion riders is also typically observed in a study among adults in New Delhi [20]. Use of a dupatta by women has been a known risk factor for RTAs in the Indian subcontinent [21]. The long dupatta (scarf) used by pillion female riders gets entangled in the rear wheel [21]. Helmet nonuse among teenage motorcyclists increases the risk of severe head injury by about 15-33 times [5]. Between 3% and 15% of all injured children in RTA are child cyclists [5]. Pedestrian children aged between 5 and 14 years have the highest risk of injury in low- and middle-income countries and account for 30%-40% of all child RTAs [5,22]. Additionally, child pedestrians are particularly vulnerable due to their limited cognitive and perceptual development, making them less adept at judging traffic speed and distance [23]. This vulnerability is compounded by inadequate pedestrian infrastructure and a lack of enforcement of traffic laws in many regions, further exacerbating the risk for child pedestrians [24].

The psychological consequences of RTAs in children are a necessary but often overlooked consideration [24]. The overall prevalence of post-traumatic stress disorder in children is at present estimated at just less than 10% [24]. In a cohort study among injured patients in North India, EQ-5D-5L utility and visual analog scale (VAS) scores were assessed longitudinally following road traffic crashes and other injuries [15], whereas in the present study, the highest mean severity index was observed for lower limb injuries (72; 95% CI = 66-77). This divergence underscores the need for region-specific injury burden assessments and HRQoL valuations to accurately inform public health interventions. Future studies should delve deeper into the long-term functional outcomes and HRQoL for child trauma patients in India, particularly focusing on the differential impacts across various injury mechanisms and socioeconomic contexts.

Adult interventions to reduce RTA, including speed limits, helmet use, and seatbelt usage, are well established and have demonstrated effectiveness in reducing morbidity and mortality [25]. These interventions can also be effective for young children, as they are often pillion passengers with adults, and for adolescents [25]. However, there are also unique aspects of childhood RTAs that require specific, targeted interventions, such as those addressing pedestrian safety and safe transport of children in vehicles. For instance, targeted behavioral interventions are needed to address factors like unaccompanied child travel, playing in streets, and nondesignated street crossing, which are significant contributors to pedestrian injuries in children [26].

The study has certain limitations. The EQ-5D-5L also has a VAS that records the overall self-rated health status on the day of the interview [27]. VAS was not used because children found it difficult to interpret. As the EQ-5D-5L is not validated in India, we reported the ICC for a subset of children. The study was done in a limited geographical area; therefore, there is a need to replicate the study in a larger population. We did not evaluate the mental health among individuals with various levels of injury, which would have helped us in correlating the HRQoL scores with the mental health of the RTA victims.

## Conclusions

The study highlights that EQ-5D-5L can be used to monitor HRQoL in children post-RTA. The results of EQ-5D-5L correlate with the injury severity. EQ-5D-5L severity index can prospectively monitor HRQoL in children that are victims of RTA. Specifically, the findings indicated that pain and discomfort were the most severe problems reported by children, with lower extremity injuries showing the highest mean severity index. The predominance of injuries from motorcycles and bicycles, coupled with low helmet usage, points to critical areas for intervention. These results emphasize the urgent need for targeted road safety initiatives and comprehensive rehabilitation programs to address the specific burdens faced by pediatric RTA victims in India. The prevalence of injuries associated with specific vehicle types and the critically low helmet usage underscore the urgent need for targeted public health campaigns for awareness of preventive strategies among both adults and children, along with stricter enforcement of road safety regulations. These findings provide crucial evidence to inform policymakers for resource allocation towards preventive strategies and comprehensive care pathways to mitigate the long-term impact of RTAs on pediatric health and well-being. Future research should focus on multi-center studies to enhance generalizability and longitudinal analyses to evaluate the long-term effectiveness of interventions, especially given the observed disparities in HRQoL outcomes across different injury types and regions.
